# An Overview on Exploitation of Graphene-Based Membranes: From Water Treatment to Medical Industry, including Recent Fighting against COVID-19

**DOI:** 10.3390/microorganisms11020310

**Published:** 2023-01-25

**Authors:** Cristina Lavorato, Enrica Fontananova

**Affiliations:** 1 Department of Environmental Engineering (DIAm), University of Calabria, Via P. Bucci, Cubo 44/A, 87036 Rende, Italy; 2 Institute on Membrane Technology of the National Research Council of Italy (ITM-CNR), Via P. Bucci, Cubo 17/C, University of Calabria, 87036 Rende, Italy

**Keywords:** antimicrobial activity, antiviral activity, COVID-19, graphene, graphene oxide, membrane, water purification, virus

## Abstract

Graphene and its derivatives have lately been the subject of increased attention for different environmental applications of membrane technology such as water treatment and air filtration, exploiting their antimicrobial and antiviral activity. They are interesting candidates as membrane materials for their outstanding mechanical and chemical stability and for their thin two-dimensional (2D) nanostructure with potential pore engineering for advanced separation. All these applications have evolved and diversified from discovery to today, and now graphene and graphene derivatives also offer fascinating opportunities for the fight against infective diseases such as COVID-19 thanks to their antimicrobial and antiviral properties. This paper presents an overview of graphene-based 2D materials, their preparation and use as membrane material for applications in water treatment and in respiratory protection devices.

## 1. Introduction

The COVID-19 pandemic was the defining global health crisis of our time—a novel infectious disease caused by severe acute respiratory syndrome coronavirus 2 (SARS-CoV-2) [1,2,3]. This type of coronavirus is an enveloped virus with a positive-sense, single-stranded RNA genome of ~30 kb. All betacoronavirus such as SARS-CoV and Middle East respiratory syndrome coronavirus (MERS-CoV), and also SARS-CoV-2, cause enzootic infections in birds and mammals that have a remarkable ability to cross species’ barriers and transmit between humans [4].

Infectious diseases caused by viruses threaten the health of both humans and animals, but often, viral diseases can be alleviated or even eradicated by vaccination [5]. In the case of COVID-19, at this time there are some vaccinations that can be used to partially protect people from this virus. In addition, the use of face mask wearing can reduce the spread of the virus because masks interfere with the virus transmission process at a level of a physical barrier independent of coronavirus variant [6]. Interesting materials that can be used to prepare mask filters are graphene and graphene derivatives, for their antibacterial and antiviral properties but also for their selective transport properties [7]. The potential of graphene could help researchers in the fight against COVID-19 [8].

**Figure 1:**
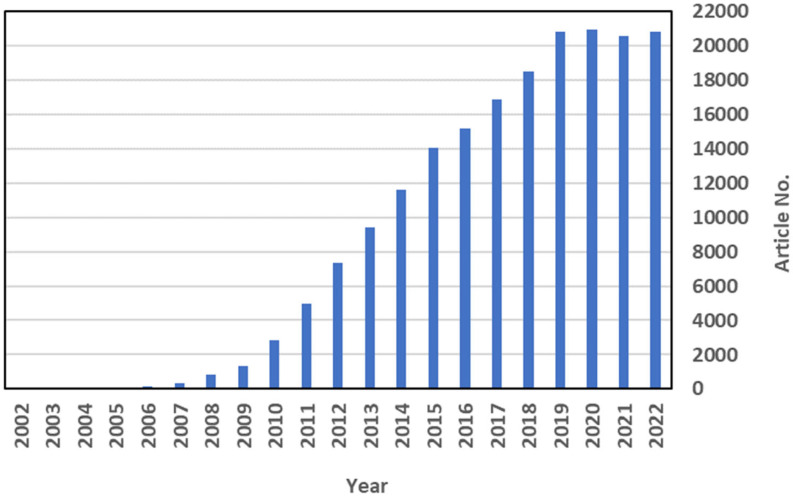
Number of articles (Article No.) regarding graphene from the year 2002 to 2022 documented in the Scopus database (keywords: “graphene”) on 28 December 2022.

Graphene (G) is a carbon sheet one atom thick and made up of condensed six membered rings. It is a two-dimensional allotrope of carbon, separated from graphite for the first time in 2004 by A. Geim and K. Novoselov who were awarded the 2010 Nobel Prize in physics for this discovery [9]. Single-layer graphene has attracted a lot of research interest due to its unique electrical, mechanical, optical, and thermal properties and chemical stability [10]. The interest in graphene-based materials for different applications is constantly increasing; it started with very few publications on graphite in 1991 then increased with the delamination of graphite in graphene sheets. The graph shows the growing number of articles on graphene in the last 20 years (Figure 1). We suppose that the number of publications on “graphene” has remained constant since 2019 because the COVID-19 pandemic may have impacted the scientific production of non-COVID-19 research. This hypothesis is supported by the meta-research gathering publications carried out by Raynaud et al., which highlighted that a rise in COVID-19 publications was accompanied by a decrease in non-COVID-19 research in the medical field [11].

Graphene-based materials are widely applied in green energy such as fuel cell, solar cell, lithium-ion batteries (LIBs), supercapacitor, dye-sensitized solar cells (DSSC), photocatalysis and photo-electrochemical cells for hydrogen evolution via water splitting [12]. Reduced graphene oxide (rGO) and graphene oxide (GO), are used for many different applications [13]: as semiconductors in photocatalytic reactions [14]; as a membrane for selective separation of different molecules or salts [15]; to produce graphene-based wearable e-textiles to reinforce fiber properties; and for improving some performance such as thermal conductivity. The combination of graphene with the polymer can improve the mechanical, electrical and thermal stability of the polymeric fibers [16,17], and is a promising chemical platform for various biomedical applications [18] including protein adsorption, bacterial and antiviral disinfection [18,19], bioreduction [20], biosensors [21] and dental application [22,23]. 

Graphene oxide and its derivatives, such as others nanoscale materials, have been widely explored for their antimicrobial properties due to their high surface-to-volume ratios and unique chemical and physical properties [24,25]. On the contrary, the literature is poor on their effects on viruses [26]. The possibility of using graphene oxide in bioengineering applications is due to the apparently low toxicity, the oxygen-containing functional groups and the unique physicochemical properties.

This review, after an initial overview on application of graphene-based membrane for water purification, focuses on the antibacterial and antiviral activity of graphene-based materials and the possibility of using graphene-based membrane for reusable and recyclable masks.

## 2. Brief Overview on Graphene and Its Derivatives

Graphite is a 3D material that is organized and built up by millions of graphene layers. Graphene (G), being one-atom thick layer of extended sp2 carbons, represents the limit of the thinnest possible 2D conductive surface, exhibiting very fast electron mobility and high charge carrier density [27]. Until now, various methods have been developed for the synthesis of graphene. Among them, three synthetic approaches have been adopted frequently: (1) Chemical Vapor Deposition (CVD); (2) mechanical cleavage from natural graphite; and (3) chemical methods. The CVD method is very useful for pure and single-layer graphene production in which the number of layers and defects in graphene can be controlled. 

Other derivatives of graphite are graphene oxide (GO), reduced graphene oxide (rGO), N-doped G (NG), graphene quantum dots (GQDs) etc. [28]. One of the most convenient procedures for the preparation of Graphene oxide (GO) suspensions consists in the deep oxidation of graphite flakes by three main synthesis routes followed by exfoliation [29].

**Figure 2:**
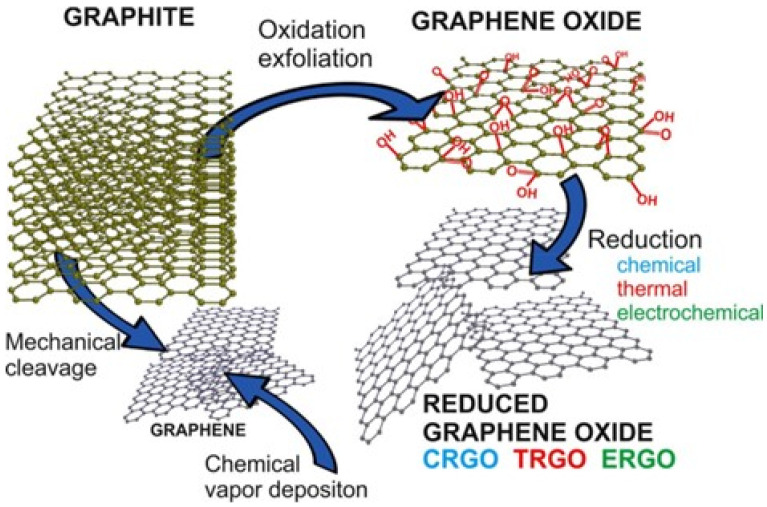
A schematic illustration of possible ways for preparation of G, GO and rGO. Reprinted with permission from ref. [34].

The most common oxidation technique used is the Hummers method [30,31], where a strong oxidant KMnO_4_ is used for graphite oxidation in the presence of H_2_SO_4_, NaNO_3_, and H_2_O_2_. The second is the Brodie method that is performed by adding potassium chlorate to slurry of graphite in fuming nitric acid [32]. The last is the Staudenmaier method that uses concentrated sulfuric acid in combination with fuming nitric acid and KClO_3_ [33]. Depending on the type of oxidants used, on the graphite source (natural or synthetic graphite flakes) and reaction conditions, the products of these reactions show strong differences in terms of different lateral sizes (from several nanometers up to several micrometers) or the composition of GO structures that affect physical, chemical, optical, and electrical properties of the nanosheets. The resulting graphene oxide can be reduced by using different methods to obtain Reduced graphene oxide (rGO) [34]. Figure 2 shows two ways to prepare G (mechanical cleavage and CVD): the GO preparation by oxidation exfoliation of graphite and the GO reduction to rGO.

The advantage of the surface oxygen functionality of GO is its hydrophilicity and the possibility of further functionalization, to form nanocomposites or to deposit as thin films on different substrates [29]. In fact, GO contains a large number of oxygen-containing functional groups such as hydroxyl and epoxy functional groups localized on the basal carbon lattice, and carboxyl groups at the edges that are active sites for functionalization and hybridization using other materials such as metal ions and metal oxides [35]. Moreover, the possibility of large-scale production of GO nanosheets makes them inexpensive and easy to study.

An interesting sustainable alternative in the approach of the synthesis of G and its derivatives is the biomass conversion that is an inexpensive source [36]. During the last 10–15 years several productive engineering approaches have been developed for the transformation of biomass waste into carbon nanomaterials by pyrolyzing via different reaction mechanistic. An example of biomass valorization is the transformation of chitosan, a natural nitrogen-containing biopolymer that is one of the main solid wastes from the fishing industry and widely available, into N-doped Graphene and graphitic nanoplatelets denoted as (N)G [37]. Preparation of (N)G was accomplished by pyrolysis under an inert atmosphere of chitosan aerogel beads and exhaustive sonication in water, leading to, after the removal of the residues by centrifugation, a persistent well-dispersed (N)G suspension without the need of surfactants or any other additive.

### 2.1. Graphene-Based Membranes 

Membrane science and technology play an important role in chemical and environmental industries with a wide range of applications [38]. In general, a membrane can be defined as a selective barrier with pores or channels to separate efficiently specific molecules and ions from mixtures in a selective way. Currently, polymeric membranes have dominated the global membrane market, such as water purification, desalination, vapor/gas and gas separations, because of their simplicity, ease of use, compact modular nature, low cost and energy efficiency, but they also have some limitations about permeability and selectivity. Therefore, membrane scientists are still looking for new membrane materials to improve membrane performance. Graphene and its derivatives are interesting membrane materials because of their outstanding mechanical and chemical stability and because graphene is the thinnest two-dimensional material with potential pore engineering [39]. Defect-free graphene is impermeable to gases or ions that try to permeate the graphitic basal plane due to the high electron density of the aromatic rings that repel any atoms or molecules. Membrane based on graphene can have high productivity and permeate permeance due to the very low atomic thickness.

Graphene-based materials are the most studied for the preparation of nanoporous membranes, multilayered membranes and mixed-matrix membranes [40,41]. The water permeability of nanoporous graphene resulted several orders higher than that of conventional reverse osmosis membrane by molecular dynamics simulations [42]. Moreover, graphene-based materials have been used to modify conventional separation membranes for ultrafiltration (UF) [43], nanofiltration (NF) [44] reverse osmosis (RO) [45], forward osmosis (FO) [46], ion exchange [47], pervaporation [48], and gas separation [49] to improve the separation. Furthermore, multilayered GO membranes can overcome membrane fouling. Their separation performance can be controlled by changing GO preparation, e.g., modifying the oxidation degree and the presence of functional groups. The GO surface is decorated with hydroxyl, epoxy and carbonyl groups that minimize aggregation in solution state due to electrostatic repulsions between GO sheets. Furthermore, these functional groups can be valuable reactive sites for further surface modification and make GO hydrophilic, which enables easy dispersion in aqueous solution by further sonication (GO contains both hydrophilic and hydrophobic domains) [50]. An important parameter to be considered during GO membrane preparation is the degree of oxidation of GO that can influence the layer-to-layer distance (d-spacing) of the GO sheets. In fact, the d-spacing has a function to exclude small molecules on the basis of their size and to provide channels for water transport. The thickness of pristine graphene is 0.34 nm [51]; this space can be improved up to about 1 nm increasing the oxidation degree [52], but also water or some humidity conditions can expand the GO nanosheets d-spacing [53]. The most common types of GO membranes preparation methods are: (i) the vacuum/pressure-assisted self-assembly method; (ii) casting/coating method; and (iii) layer-by-layer method [54].

Vacuum-assisted and pressure-assisted methods are both filtration processes used to deposit GO nanosheets near and almost parallel between them to a support. The thickness of GO membranes prepared change with the volume or concentration of the GO dispersions. In general, this method is used to prepare microfiltration and ultrafiltration membranes. Another type of preparation is the evaporation-assisted method that was found to produce GO membranes with higher surface roughness and water contact angle to compare with the microstructures of GO sheets prepared by the pressure-assisted methods that exhibited a highly ordered laminate structure and the lowest surface roughness [55]. High rejection and flux were found with GO membranes prepared with a high order degree of GO sheets [56].

Casting and coating are methods used mainly to prepare freestanding GO membranes and can be used with a wider range of substrate, e.g., copper foils by a coating method, smooth paper by drop-casting, and dip-coating to fabricate hollow-fiber membranes [40]. The layer-by-layer (LBL) self-assembly method consists of assembling oppositely charged polyelectrolytes to prepare a thin film charged at the molecular level. The process of LBL assembly is random and for this reason the membrane surface obtained is very rough. In general, polycations and polyanions are used as assembly material for LBL membranes, which can be deposited on the substrate, before modification to form a charged surface, through electrostatic interactions [57]. The LBL self-assembly method can also be used to assemble two nanomaterials into a multilayered structure. To fabricate LBL GO membranes an electric field and crosslinking were also used [58]. 

The simplest graphene-based desalination membrane can be produced by making nanoscale pores in a layer of graphene [15]. To produce porosity on one-atom-thick graphene membrane with very small diameter, two typical methods can be used: (a) top–down method that includes hole drilling by high-temperature oxidation, electron beam, UV irradiation or plasma or helium ion bombardment; and (b) bottom–up with direct chemical synthesis from monomer (e.g., self-assembly via Ag-catalyzed polymerizations) [38]. O’Hern et al. reported the synthesis of graphene membrane with a pore size that allows selectivity of molecule separation while still maintaining high water permeability [59]. These pores were created into the lattice of CVD graphene through initial Ga ion bombardment with acceleration voltage of 8 kV and density of 6 × 1012 ions cm^−2^, and subsequently they are enlarged by chemical oxidative etching based on acidic potassium permanganate. The final permeable pores had the diameter of 0.40 ± 0.24 nm.

Although, single-layer graphene membranes have shown potential as a reverse osmosis (RO) desalination membrane, from simulation work their practicality is limited because of the lack of robustness required for real-world applications [42]. Instead, the synthesis of multi-layer graphene-derived membranes is more economical than the production of single-layer membranes, with similar properties as single-layer membranes [60]. 

Ultrathin self-standing GO membranes were reported to have unexpected performance in separation processes thanks to the 2D nanostructured interlayer space between GO layers [51,61,62]. However, the great potential of GO membranes is restricted by their limited stability in aqueous solutions due to swelling and delamination phenomena [63]. Numerous approaches were investigated to increase GO membrane stability [64].

Moreover, additional issues limiting large-scale applications of GO-based membranes are the complexity of the membrane preparation protocols and difficulties in scale up.

**Figure 3:**
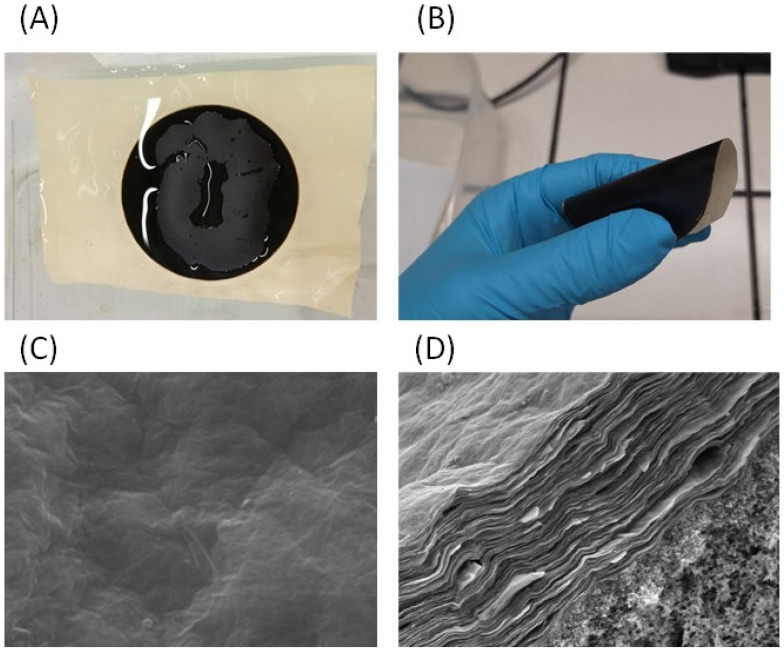
Composite GO membrane supported on the crosslinked co-poly(amide-imide) membrane. Pictures of the membrane (**A**) in wet and (**B**) dry state. SEM images of (**C**) the surface and the (**D**) cross section. Reprinted with permission from ref. [65].

An easily scalable method with a low environmental impact exploiting GO self-assembly on a porous co-poly(amide-imide) (PAI) film driven by attractive electrostatic interactions was recently developed to obtain GO membranes with excellent stability, both in wet and dry state (Figure 3A,B) [65]. 

The membranes present a composite structure with 2D layered GO supported on macroporus PAI (Figure 3C,D). The experimental results highlighted the key role of support chemistry, surface charge and topography to obtain stable composite membranes. These GO-PAI membranes maintained an interlayer spacing of ∼9 Å after long-term storing in water or saline solution, and were able to reject organic dye with a water flux more than 1000 times higher than predicted for a viscous flux through such GO model membrane [64]. Modelling activities provided the details of the comprehension of the relations between the membrane support and the GO selective layer [66]. 

Moreover, new PVDF-GO mixed matrix membranes were also developed using DMSO as non-hazardous solvent, in place of traditional substances of very high concern (SVHC), in a combined vapour- and liquid-induced phase separation process in which water in vapour or liquid form works as a non-solvent for the polymer inducing its precipitation. The protocol developed did not include any chemical additive generally used as pore former (e.g., LiCl, PVP) in phase separation process for the production of porous membranes [67]. The presence of the 2D-nanofiller in the casting solution influenced surface charge and also the microstructure of the formed membranes. The PVDF-GO mixed matrix membranes were characterized by a spherulitic morphology with high surface roughness. They had improved rejection and similar flux in comparison with polymeric PVDF membranes prepared by the same protocol but without the GO. Moreover, PVDF-GO resulted less prone to fouling than PVDF membranes [67].

### 2.2. Graphene-Based Membrane for Water Purification

Contamination of water with bacteria, protozoans and viruses is a serious problem because water acts as a carrier for infectious pathogens that spread harmful diseases [68]. 

The literature available regarding the separation of viruses from the water mostly denotes the use of polymeric membranes depending on their pore size [69]. In fact, to obtain a complete rejection of a virus ultrafiltration membrane is usually required. Some experimental investigations reported the complete removal of virus with the UF membranes having pore size 0.2 nm [70,71]. There are two main groups of viruses commonly found in wastewater [72]. Firstly, viruses that are more resistant to natural and engineered inactivation processes (e.g., UV from sunlight and UV treatment systems, respectively) and have an external protein capsule, but not a lipid envelope, such as DNA viruses (e.g., adenovirus), as well as RNA viruses (e.g., hepatitis A, E viruses, norovirus and other enteroviruses, etc.). The second group of viruses found in wastewater include the influenza virus, coronaviruses (e.g., COVID-19) and the herpes virus, which have a lipid envelope and are more fragile. Some authors reported that this type of virus can be detected in feces, but they are not typically associated with waterborne disease transmission and outbreaks [73].

Qu et al. reported that COVID-19 can survive in stool samples for 4 days and that this virus has been positively detected in stool samples of infected patients [74]. In other studies, coronavirus was reported to remain in water and sewage for days to weeks [74,75,76]. Ransome et al. reported that infectious SARS-CoV-2 inoculum is stable in Thames water and sediment for <3 days, while SARS-CoV-2 RNA is detectable for at least seven days [77]. 

Membranes for water purification and water desalination are being used more and more to address global challenges of pollution and scarcity of water [78]. Highly selective and high permeable next-generation membranes are proposed to address the limitations of the current membrane technologies. Graphene derivatives are among the most promising of several materials. To limit the water pollution by some pathogens the possibility of using graphene and graphene derived materials can be considered [75].

Some authors reported that the antibacterial ability of graphene can be increased when doped with metal nanoparticles [79], because silver suppresses the DNA replication process of the bacterium [80]. However, the silver nanoparticles’ efficiency is dependent on different factors including their size, shape, co-composite materials, coating thickness/morphology and concentration [81,82]. Moreover, titanium dioxide, zinc oxide and magnesium dioxide nanoparticles have demonstrated antimicrobial activity such as graphite and graphene that include damage of cellular membranes, oxidization of cellular components and disruption of normal microbial processes via direct contact with microbial cells [83]. Indeed, GO degrades the outer and inner membrane of *E. coli* and creates strong interactions between itself and lipid molecules in bacteria [84]. 

Magnetic graphene-based nanocomposites are used as an antibacterial agent for water purification [85,86]. Both graphene and carbon nanotube CNT were combined independently with silver nanoparticles to obtain nanomaterials, which exhibited enhanced antibacterial activity against two strains of infectious bacteria such as *Escherichia coli* (*E. coli*, Gram-negative) and *Staphylococcus aureus* (*S. aureus*, Gram-positive) [87]. These combined materials were effective in controlling biofouling [88]. The large surface area of carbon-based materials and high catalytic activity of silver nanoparticles can be interesting to disinfect by infectious pathogens. Similar results were also observed in inactivation of *E. coli* using recycled graphene oxide–iron oxide–silver nanocomposites [89]. Other studies will be reported in paragraphs 3 and 4 focused on antibacterial and antiviral activity, and this concept has potential for water treatment and for the preparation of respiratory devices to protect persons from infected people against coronavirus disease.

## 3. Antibacterial Activity of Graphene-Based Materials

The growing antibacterial resistance spectrum of bacterial infections required the development of novel and efficient antibacterial agents. Biocidal nanomaterial is usually used in the science field to improve medical devices’ wastewater treatment, food packaging, synthetic textiles and dentistry [90]. 

The increased attraction towards graphene and graphene derivatives such as graphene oxide and reduced graphene oxide is determined by the bactericidal activity of G on several Gram-negative and -positive bacteria [91]. G is one of the most recently developed biocidal nanomaterials for its robustness, mechanical strength, great surface area, and resistance to degradation due to its superior surface-to-volume ratio [92,93]. The attachment and adherence of G to biological cells is due to their active segments, edges and surfaces. In particular, the degree of antibacterial effect of nanomaterials is determined by their shape, surface functionalization, size, stability and size distribution [90,94].

Sometimes authors reported different behavior of graphene derivatives in terms of antibacterial effect. Liu et al. compared the antibacterial activity of graphite (G), graphite oxide (GtO), graphene oxide (GO), and reduced graphene oxide (rGO) toward a bacterial model such as *Escherichia coli* investigating its antimicrobial mechanism [95]. The authors reported that GO dispersion shows the highest antibacterial activity, successively followed by rGO, G, and GtO. Contrariwise, another study on antimicrobial activity of graphene oxide showed that in this case bacteria were able to attach and proliferate in the presence of GO indicating a lack of antibacterial properties [96]. Other studies showed dissimilar performance of GO in terms of inhibition effect that depends on the type of bacteria. For example, GO and reduced GO can lead to the fragmentation of bacterial DNA and death by producing reactive oxygen species in Pseudomonas. GO-Ag can disrupt the cell wall of Gram-positive bacteria and inhibit cell division of Gram-negative bacteria [97]. De Faria et al. reported that GO sheets showed no antibacterial activity, while GO-Ag had a 100% inhibition rate of the adhered cells [98]. These opposite results are probably due to the different interaction mechanisms between GO and pathogens [98]. Many authors reported that the interaction between GO and bacteria are affected by the morphology, GO size, concentration, exposure time, incubation protocol, and microorganism type [35,99]. The antibacterial efficacy of GO is mainly in the form of freestanding paper, nanosheets, or nanowalls. Three main mechanisms of actions were suggested: nanoknives due to sharp edges; oxidative stress; and the wrapping or trapping of bacterial membranes by flexible, thin GO films. Well-dispersed, freestanding GO nanosheets have been reported to have the highest antibacterial activity among several G-based nanomaterials. In some cases the reactive oxygen species (ROS) mechanism was considered as the principal factor of antibacterial activity [100]. For example, GO at a perpendicular position to the bacteria could exhibit higher antibacterial activity against *Escherichia coli* than against *Staphylococcus aureus* because of the absence of any additional outer membrane in the case of *S. aureus*. *E. coli* was more damaged by the GO coatings than *S. aureus*, probably for the oxidative stress from GO [101]. 

**Figure 4:**
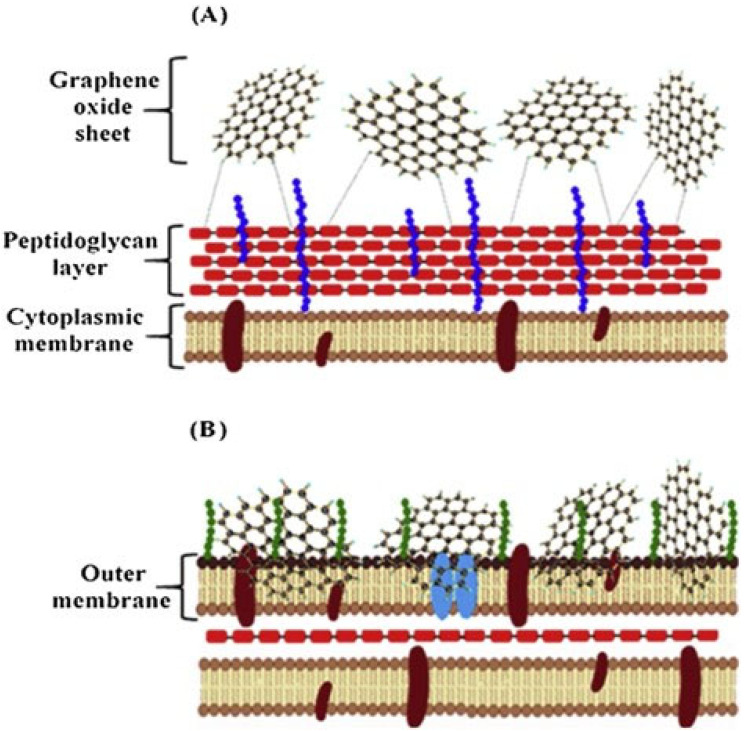
Design of the possible mechanism of GO interactions with Gram-positive and Gram-negative bacteria. (**A**) Mechanical wrapping in Gram-positive bacteria and (**B**) the damage of the membrane in Gram-negative bacteria. Reprinted with permission from ref. [102].

Investigating the interactions of GO with Gram-positive and Gram-negative bacteria, often the antimicrobial effects of GO are concentration and time dependent. The main structural difference between these two types of bacteria is that Gram positive bacteria have a thick peptidoglycan layer and no outer lipid membrane, while Gram negative bacteria have a thin peptidoglycan layer and have an outer lipid membrane. Figure 4 represents possible interactions of GO with membranes of Gram-positive and Gram-negative bacteria strains [102]. Figure 4A shows a mechanical wrapping in Gram-positive bacteria and Figure 4B shows the damage of the membrane in Gram-negative bacteria.

Wang et al. [103] studied the antibacterial activity and cytotoxicity of ZnO/GO composites against *E. coli* strain and human HeLa cells. The typical bacterium *Escherichia coli* and HeLa cell were used to evaluate the antibacterial activity and cytotoxicity of the ZnO/GO composites, respectively. The antibacterial activity of the ZnO/GO composites is dependent on the content of zinc in the composites. It is mainly due to the synergistic effect of ZnO and GO: the possibility of bacteria contact to zinc was increased as ZnO NPs and released zinc ions from ZnO NPs were enriched on the GO sheets. The intimate contact of the *E. coli* cells and ZnO NPs on the GO sheets enhanced the permeability of the bacterial membrane and the local free zinc concentration around bacteria. The cell viability loss induced by the composites is dose-related. GO exhibited no viability loss, even at relatively high concentrations (50 μg mL^−1^), indicating the low toxicity of GO to HeLa cells. Similarly, even when the concentration of ZnO/GO-1 (prepared with mass ratio of ZnO/GO was 3:1) reaches 20 μg mL^−1^, the cell viability is still over 90%. However, a concentration of ZnO NPs (240 μM) decreased the cell viability to 30%. These results illustrate that the ZnO/GO composites cause much lower cytotoxicity than an equivalent amount of ZnO NPs alone. In another study [104], ZnO/graphene composite showed a 100% inhibition of *E. coli* after 12 h. This antibacterial activity was probably caused by the synergy of the physical interactions of graphene with bacterial membranes and the antibacterial properties of ZnO that have the aptitude to photo catalytically generate H_2_O_2_ and to penetrate cells and disrupt the bacterial membrane. Furthermore, graphene oxide can be easily modified with silver NPs, obtaining the advantages of the physical properties of GO and the strong antibacterial activity of Ag NPs [105]. Some researchers showed exceptional antibacterial activity against the Gram-negative bacterial strain *P. aeruginosa*. De Faria et al. [98] reported that the GO dispersion showed no antibacterial activity against *P. aeruginosa* over the concentration range investigated. While the GO–Ag nanocomposite showed an antibacterial activity with a minimum inhibitory concentration ranging from 2.5 to 5.0 μg/mL. Moreover, the results showed a 100% inhibition rate of the adhered *P. aeruginosa* cells on stainless steel surfaces after exposure to the GO–Ag nanocomposite for one hour. This interesting result showed the possibility of using GO–Ag nanocomposites as antibacterial coatings material to avoid the growth of biofilms in food packaging and medical devices.

Lukowiak et al. [29] reported various antibacterial mechanisms of action for graphene-based structures. In addition to the mechanism of the membrane cell disruption (cell damage mechanical), graphemic-based materials can: (i) wrap around the bacteria isolating them from the environment; (ii) produce damaging reactive oxygen species (ROS); (iii) extract phospholipid molecules of the bacteria membrane due to the lipophilic graphene; and (iv) reduce the metabolic activity of the bacterial cells. ROS production is the most cited factor to kill bacteria by GO materials. 

**Figure 5:**
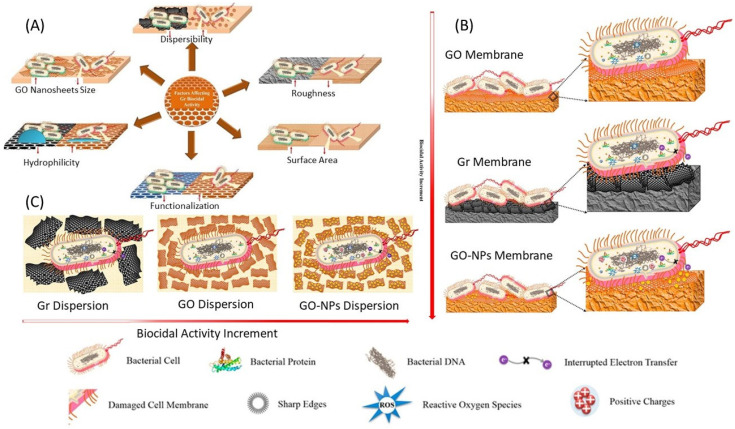
(**A**) The physiochemical properties of graphene nanomaterial relevant to its contradictory antibacterial effect. (**B**)Schematic diagram illustrating the antibacterial strength of different G-based suspensions. (**C**) Schematic diagram illustrating the antibacterial strength of different G-based surfaces. Reprinted with permission from ref. [90].

The interaction of G-based nanomaterials with bacterial cells depends on their sheet size, surface area, roughness, hydrophilicity, dispersibility and functionalization [90]. The separate effect of each factor on the viability of bacterial cells is illustrated in Figure 5A. The antibacterial activity of G-based materials was observed to be influenced by the size of the GO sheet. Smaller sheet size resulted in enhanced antibacterial activity for G-based surface coatings. In general, the sharp edges, small size and rough surfaces of G nanosheets facilitate their internalization into the bacterial cells compared to larger and smooth sheets, and the antibacterial activity improved as the GO concentration increased, until 80 mg/mL (antibacterial activity >90%) [106]. Normally, bacterial cells have higher affinity for hydrophobic surfaces due to hydrophobic interactions, which depends on the type of bacteria tested. Adhesion for some bacterial species may be related also to surface chemistry. A crucial factor affecting interaction with bacterial cells is the physical structure of the surface. Bacterial cell adhesion improves when the surface roughness increases. GO has a higher bactericidal activity compared with rGO because the GO dispersion, which comprises two-dimensional carbon sheets [95], is stable due to the great number of different functional groups with hydrophilic nature, e.g., hydroxyl, carboxyl, and epoxy groups [107]. The rGO has a size approximately nine times bigger than that of GO nanosheets. Thus, sometimes they easily aggregate leading to a lower antibacterial activity of rGO particles. 

### G-Based Suspension/Membrane Antibacterial Activity

The interaction of bacteria with GO follows a different mechanism when it is in suspension form or immobilized on a coated surface [90]. The physicochemical features of GO, e.g., sheet size, can have a diverse effect when GO nanosheets are coated on a surface [108]. Some authors suggested that the bacterial cell toxicity differs according to the orientation of the sheets assembled on the surface, which can facilitate or impede the contact with bacterial cells in a way of edgewise interaction [109]. To obtain GO antibacterial activity, cellular membrane destruction has to initiate by phospholipid drawing out or nanosheet piercing by GO and it is primarily caused by orthogonal interaction with GO nanosheet edges or by a contact with basal planes [110,111]. When GO is coated on surfaces, the orthogonal cell interaction with nanosheet edges is reduced and the contact with basal planes is improved.

The biocidal strength order of Gr-based nanomaterials dispersions is reported as follows: G hybrid NPs, GO and G (Figure 5B); while the antibacterial strength of G-based nanomaterial surfaces could be summarized in the order of G hybrid NPs, G and GO (Figure 5C) [90].

## 4. Antiviral Activity of Graphene-Based Materials

Despite virus vaccination being the most widely used method for alleviating or eradicating some diseases, attenuated vaccines are not available for all viral infections [5]. The risk of emerging or re-emerging diseases entails the need to develop always novel antiviral compounds. Between various nanomaterials studied for this purpose, interesting new research is being conducted on the antiviral activity of graphene-based materials.

In recent years, graphene oxide derivatives were studied to inhibit some viruses such as herpes simplex virus type-1 (HSV-1) competing with the heparan sulfate in binding HSV-1 [112]. 

One of the major viral pathogens of the lower respiratory tract of infants is the respiratory syncytial virus (RSV). Yang et al. [26] demonstrated that the antiviral activity of a novel nanomaterial composed by curcumin and β-CD functionalized GO (GSCC) against RSV infection was dose-dependent. Moreover, GSCC could inactivate RSV prior to infection efficiently. There are three possible mechanisms for GSCC inhibiting RSV infection, including directly inactivating RSV, inhibition of the attachment of virus onto host cells and interfering with virus replication. 

### 4.1. Graphene-Based Materials against Virus of Coronaviridae Family

Very few studies have reported on the inhibition of the entry and replication of enveloped DNA virus (herpesvirus) and RNA virus (coronavirus) in their target cells by using graphene-based material [113]. Ye et al. evaluated the antiviral properties of GO and reduced GO, demonstrating that they exhibit broad-spectrum antiviral activity toward pseudorabies virus (PRV), a DNA virus, and porcine epidemic diarrhea virus (PEDV), a positive-strand RNA virus belonging to alpha-coronavirus, at a noncytotoxic concentration (6 μg/mL) [5]. PRV and PEDV are spherical viruses approximately 200 and 100 nm in diameter. The GO used in this study, with size of about 500 nm, exhibits significant antiviral properties even at a low concentration (1.5 μg/mL). After incubation with GO for 1 h, part of the envelope and spikes of mature PRV and the crown and the envelope of PEDV were destroyed. The destruction of viral morphology suggested that GO may inactivate the virus by using its sharp edge as well. In fact, in a previous study, Akhavan et al., found that GO nanowalls can inactivate bacteria by direct contact with their sharp edges [114]. Tu et al. also reported that GO nanosheets can also penetrate cell membranes of bacteria and extract phospholipids [110]. 

**Figure 6:**
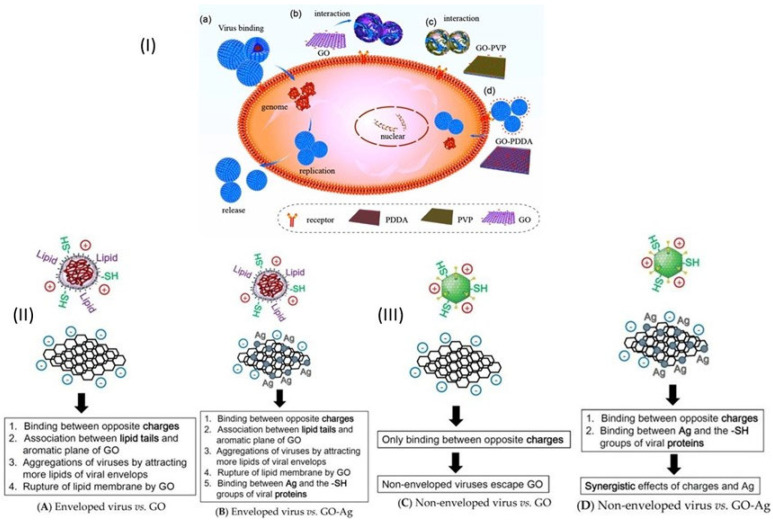
(**I**) Possible Mechanisms of the antiviral activity of GO. (**a**) Normal viruses are absorbed into cells by interacting with cell receptors to initiate infection. (**b**) Negatively charged GO has more chances to interact with the positively charged viruses, leading to virus damage and the inhibition of infection. (**c**) Infection was blocked by GO conjugated with nonionic PVP but not with cationic PDDA (**d**). Reprinted with permission from ref. [5] Copyright 2022 American Chemical Society. (**II**,**III**) Schematic for the antiviral mechanisms of (**A**) graphene oxide (GO) against the enveloped. virus; (**B**) graphene–silver nanocomposites (GO-Ag) against the enveloped virus; (**C**) GO against the non-enveloped virus; (**D**) GO-Ag against the non-enveloped virus. Reprinted with permission from ref. [113].

Ye et al. analyzed the impact of GO charge by observing the antiviral activity of GO conjugated with cationic polymer PDDA or nonionic PVP [5]. The results showed that the negative charge is required for the antiviral mechanism of GO and rGO; in fact, the nonionic GO-PVP shows similar antiviral activity as GO while the cationic GO-PDDA has no antiviral activity. However, GO inactivates the viral particles only prior to their entry into cells, outside cells GO interacts with the positively charged virus particles causing their destruction and inactivation (Figure 6I). The nanosheet structure is important for the antiviral activity; indeed, polylaminate GtO shows much weaker antiviral activity than single-layered GO and rGO, whereas the non-nanosheet Gt shows no antiviral activity. Contrariwise, the oxygen-containing group is not essential for the antiviral activity, and in fact, both GO and rGO show similar antiviral activity.

Chen et al. [113] investigated the antiviral activity of graphene oxide sheets and GO sheets with silver particles (GO-Ag) against enveloped virus, feline coronavirus, and non-enveloped, virus infectious bursal disease virus (IBDV). Feline coronavirus (FCoV) is a positive-sense, single-stranded RNA virus with a lipid envelope that belongs to the family Coronaviridae. IBDV is a double-stranded RNA virus without an envelope and belongs to the genus Avibirnavirus of the family Birnaviridae. A virus inhibition assay was used to identify the antiviral activity of GO and GO-Ag. GO-Ag inhibited 25% of infection by FCoV and 23% by IBDV, whereas GO only inhibited 16% of infection by FCoV but showed no antiviral activity against the infection by IBDV. This different antiviral activity of GO sheets against enveloped and non-enveloped viruses is probably caused by a physical or chemical interaction between GO sheets and the envelope of coronavirus. Negatively charged GO can absorb to positively charged lipid membranes and induce the rupture of lipid membranes; the ruptured lipid membrane would associate strongly to the aromatic plane of the GO sheet, increasing the absorption of more lipid membranes [115,116,117]. Although the antiviral mechanism requires further research, the authors proposed a tentative model for the antiviral mechanisms of GO and GO-Ag against enveloped and non-enveloped viruses (Figure 6II,III) [113].

A significant advantage of GO-Ag nanocomposites over free Ag NPs is the increasing biocompatibility of the material and the toxicological effects of reducing associated with metallic nanoparticles due to their immobilization on GO. GO-Ag nanocomposites have a specific surface area, are not corrosive and are highly dispersible in water. An important use of GO sheets is as a supporting and stabilizing agent in preventing the agglomeration of the Ag NPs that showed a spherical-like morphology and an average size of 7.5 nm, which is suitable for antiviral activity [105,113,118]. 

### 4.2. Gr-Based Film/Membrane Antiviral Activity

The study of graphene-based membrane with antiviral activity is still at a nascent stage. Akhavan et al., fabricated Graphene−tungsten oxide composite thin films with sheetlike surface morphology that were applied in photoinactivation of viruses under visible light irradiation [119]. The viruses on surface of the graphene−tungsten oxide composite film suffered a nearly complete destruction of the viral protein and a sharp increase in the RNA efflux after 3 h light irradiation at room temperature. The composite film showed <10% reduction in the RNA efflux after 20 measurement cycles, indicating stability in its photocatalytic performance up to 60 h irradiation. 

Graphene possesses excellent light-to-heat conversion ability owing to its superior thermal conductivity. Deokar et al. designed a graphene-based photothermal antiviral agent that captures and destroys viruses [120]. They synthesized sulfonated magnetic nanoparticles functionalized with reduced graphene oxide (SMRGO) to capture and photothermally destroy herpes simplex virus type 1 (HSV-1). Graphene sheets were uniformly anchored with spherical magnetic nanoparticles (MNPs) of varying size between ∼5 and 25 nm. Upon irradiation of the composite with near-infrared light (NIR, 808 nm, 7 min), SMRGO (100 ppm) demonstrated superior (∼99.99%) photothermal antiviral activity. This was probably due to the capture efficiency, unique sheet-like structure, high surface area, and excellent photothermal properties of graphene. In addition, electrostatic interactions of MNPs with viral particles appear to play a vital role in the inhibition of viral infection.

## 5. Respiratory Protection Devices for COVID-19

The main transmission mode of virus such as COVID-19 is through short-range aerosols and droplets [121]. Micron-sized aerosols containing the virus are released into the air from infected people when they breathe, speak, sing, cough, or sneeze. Although the actual COVID-19 virus is ca. 150 nm, data reported from influenza patients suggest that the aerosols containing the virus can be of two main type: (i) fine (<5 μm), which can stay in the air nearly indefinitely; or coarse (>5 μm), which can settle due to gravity within 1 h, or if they are >10 μm settle more rapidly [122]. 

For this reason, the use of disposable surgical masks or respirator are common for patients, doctors, and even the people in high-risk areas. 

### 5.1. Respirators

The United States Centers for Disease Control and Prevention (CDC) recommends the usage of N95 filtering facepiece respirators (FFR) as personal protective equipment for healthcare professionals [121]. The CDC’s National Institute of Occupational Safety and Health (NIOSH) (document 42 CFR Part 84) determined the minimum filtration efficiency of 95% for 0.3 μm (aerodynamic mass mean diameter) of sodium chloride aerosols (N95), but there are also masks with higher filtration efficiencies of 99% and 99.97% which correspond to N99 and N100, respectively. NIOSH has also created grades R and P (with filtration efficiencies 95−99.97%) for oil-based aerosols (DOP). In other part of the world the equivalent filtration grades to N95 are FFP2 (European Union), KN95 (China), DS/DL2 (Japan), and KF94 (Republic of Korea).

**Figure 7:**
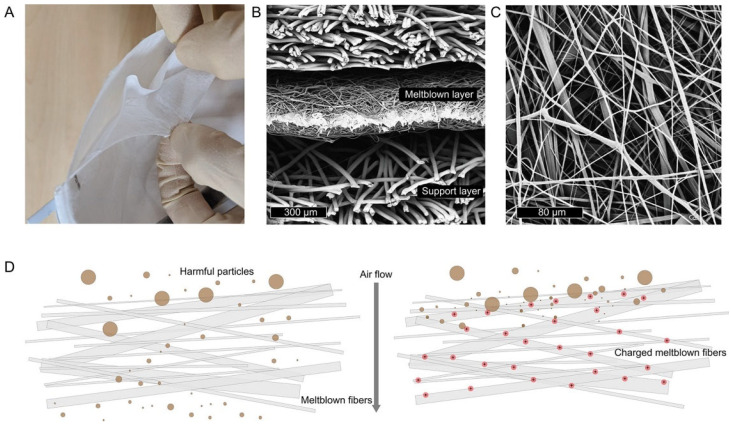
Meltblown fabrics in N95 FFRs. (**A**) Peeling apart a representative N95 FFR reveals multiple layers of nonwoven materials. (**B**) Scanning electron microscope (SEM) cross-section image reveals the middle meltblown layer has thinner fibers with thickness around 300μm. (**C**) SEM image of meltblown fibers reveals a complicated randomly oriented network of fibers, with diameters in the range of ∼1−10 μm. (**D**) Schematic illustration of meltblown fibers (left) without and (right) with electret charging. In the left figure, smaller particles are able to pass through to the user, but particles are electrostatically captured in the case of an electret (right). Reprinted with permission from ref. [121].

The N95 FFR is composed of multiple layers of polypropylene with a meltblown layer that is 100−1000 μm in thickness with polypropylene microfibers’ diameters in the range of ∼1−10 μm [121]. These fibers are charged through corona discharge and/or triboelectric means into quasi-permanent dipoles called electrets to improve the filtration efficiency. Figure 7 shows meltblown fabrics in N95 FFRs that are constituted by multiple layers of nonwoven materials (Figure 7A), with the middle meltblown layer that has thinner fibers with thickness around 300μm (Figure 7B) and diameters in the range of ∼1−10 μm that are randomly oriented. Figure 7D shows a schematic illustration of meltblown fibers (left) without and (right) with electret charging; in this last case particles are electrostatically captured. 

### 5.2. Surgical Masks

The use of disposable surgical masks is common for patients, doctors, and for all people in highly risky areas [123]. As explained before, respirators should reduce inhalation exposure to airborne particles by at least 95% if worn properly. However, with the significantly high cost of respirators and the related manufacturing, fit testing, comfort and compliance, there has been growing difficulty with the use of surgical masks to prevent droplet-borne respiratory infection; this became necessary despite the surgical masks lacking the air tightness compared to respirators [124,125]. In fact, surgical masks are usually designed to prevent droplet transmission from health care workers to surgical patients, or blood-borne infection from patients to health care workers during medical procedures, but they are generally considered to be ineffective in preventing airborne infection. However, the above-mentioned masks cannot be used for a long time [121]. The high economic and environmental costs of these non-reusable masks damages societies worldwide, and for this reason some authors reported methods to self-sterilize for reuse or to recycle masks for further use. 

In general, the mechanisms of disinfection or sterilization of bacteria and viruses include protein denaturation (alcohols, heat), DNA/RNA disruption (UV, peroxides, oxidizers), and cellular disruption (phenolics, chlorides, aldehydes), but these methods can modify the masks’ filtration efficiency [121]. Liao et al. studied the following procedures to reuse FFRs: (1) heat >70 °C for 30 min under various humidity; (2) steam at 100 °C heat for 10 min; (3) 75% of alcohol aqueous solution; (4) diluted chlorine-based solution; and (5) ultraviolet germicidal irradiation (UVGI) at a wavelength of 254 nm with an intensity of 8 W for 30 min [121]. The results showed that by using ethanol and chlorine-based solution the filtration efficiency of FFRs was drastically degraded to intolerable levels, prolonged treatment by steam to disinfect the N95 respirator decreased slowly their filtration efficiency, and contrariwise the UVGI method and heat treatment at 75 °C does not modified the filtration properties within 10 treatments, but the penetration of UV depth might be limited. 

### 5.3. Reusable and Recyclable Graphene Masks

**Figure 8:**
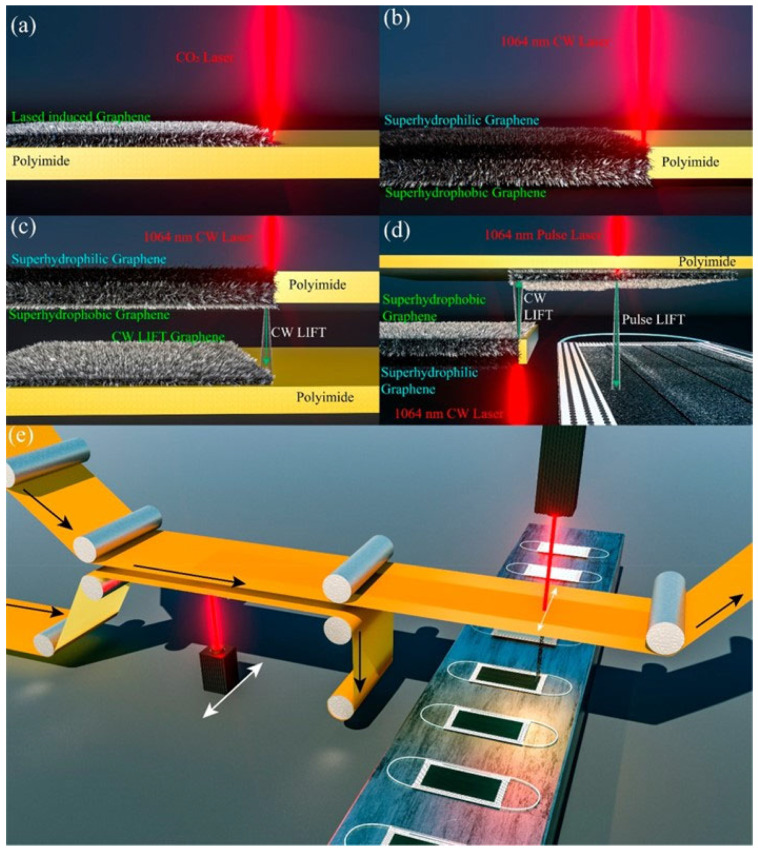
Comparison of different laser-induced graphene methods. (**a**) First-generation direct laser-induced graphene on a polyimide surface using a CO_2_ laser. (**b**) Second-generation Janus superhydrophobic/superhydrophilic surface by 1064 nm laser scribing on polyimide. (**c**) Third-generation CW-LIFT of graphene from polyimide to acceptor polyimide. (**d**) Fourth-generation dual-mode LIFT, with CW-LIFT transfer of graphene onto a second polyimide and then pulse-LIFT transfer of graphene onto a temperature-sensitive mask. (**e**) Illustration of the compatibility of the dual-mode LIFT for roll-to-roll production of a graphene-coated mask. The roll movement direction is shown as the black arrow, and the laser movement direction is indicated by the white arrows. Reprinted with permission from ref. [123]. Copyright 2022 American Chemical Society.

To limit the above-mentioned problem various authors reported the synthesis of different types of washable, sanitizable and reusable masks to protect people from infection of Coronavirus disease [123,126]. Graphene can be used to modify commercially available surgical masks to obtain self-cleaning and photothermal properties [123]. A dual-mode laser-induced forward transfer method was developed for depositing few-layer graphene onto low-melting temperature nonwoven masks. The melt-blown fibers of non-modified surgical masks have a diameter around 20 μm; the surface of these fibers is smooth, with a contact angle of approximately 110°, and this type of mask is hydrophobic but not superhydrophobic. Zhong et al. reported that by using the laser-induced forward transfer of laser-synthesized graphene the surfaces for surgical masks can become superhydrophobic [123]. Figure 8 shows different types of surgical mask modification by using graphene. The first-generation laser-induced graphene via directly scribing on polyimide cannot be used to create graphene on the mask due to the remaining polyimide after CO_2_ laser scribing (Figure 8a). To create a Janus wetting status on the opposite sides of the polyimide the second-generation laser-scribed graphene using 1064 nm can be used, but with this method it is impossible to transfer the synthesized graphene to the mask (Figure 8b). Instead, the third-generation continuous wave (CW) laser-induced forward transfer (LIFT) graphene can be deposited additionally onto other substrates (Figure 8c), but the high temperature on the targeted acceptors influenced their final morphology, damaging the surgical masks. Another method that can be used is the pulse laser-induced forward transfer (pulse-LIFT) following the CW LIFT (Figure 8d). The graphene can be coated onto the surgical mask without damaging its surfaces because by using this last method it is sufficient at a low temperature. This fourth-generation laser deposition method is also compatible with a roll-to-roll system, which can be easily integrated within the existing automatic mask manufacturing production lines (Figure 8e). 

The results showed that the superhydrophobic surfaces provide better protection toward incoming respiration droplets. This mask is reusable after sunlight sterilization because the surface temperature can quickly increase to over 80 °C under solar light. In addition, this graphene-coated mask can be recycled directly for use in solar-driven desalination with outstanding salt-rejection performance for long-term use [123].

**Figure 9:**
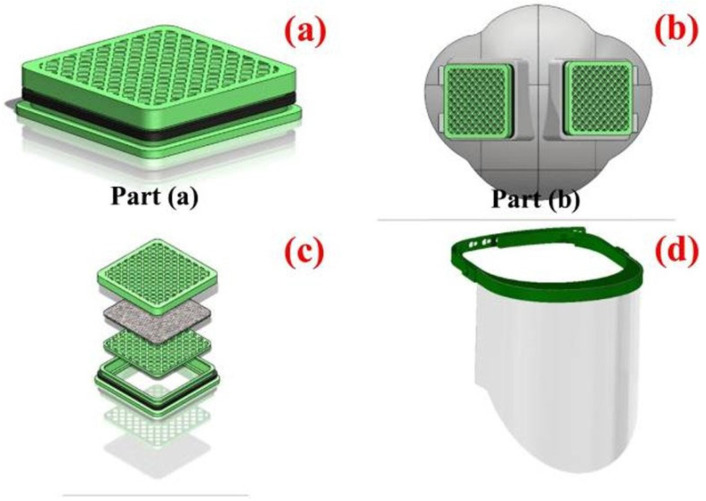
Schematic representation of the design of the nanofibrous respirator face mask. Part (**a**) depicts the respirator filter containing multilayers of CuONPs/GO@PLA and CuONPs/GO@CA nanofibers. Part (**b**) represents the fixed part of the face mask. The assembly of the multilayers consisting of nanofibers into a respirator filter is shown in (**c**). The face shield containing two parts and fabricated via the molding procedure is shown in (**d**). Reprinted with permission from ref. [127].

Recently, Ahmed et al. [127] described a novel design based on the filtration system composed of a nanofibrous matrix of polylactic acid (PLA) and cellulose acetate (CA) containing copper oxide nanoparticles and graphene oxide nanosheets, and produced using the electrospinning technique. Moreover, the flat pattern fabricated from a thermoplastic composite material is used to provide a solid fit with the facial anatomy. CuO and GO nanoparticles should inactivate viral particles caught in the membrane and then inhibit the bacterial and viral transmission by stopping the airborne viral particles in their flight. The authors produced a prototype for a customizable/multi-used protective respirator mask containing a fixed part constituted by molten PLA and a disposable part of multilayers of CA/PLA nanofibers doped with CuONPs and GO. Figure 9 shows the schematic prototype that consists of two main parts: (a) a disposable filter piece composed of unwoven nanofibers, each one constituted by multilayers of CuONPs/GO@PLA and CuONPs/GO@CA; (b) a fixed piece washable with water that could be sterilized using an UV lamp (λ = 250 nm); (c) the assembly of the multilayers consisting of nanofibers into a respirator filter; and (d) the face shield containing two parts and fabricated via the molding procedure.

Huang et al. identified the bacteria viability on common face masks and found that the majority of bacteria (90%) remain alive after 8 h [128]. The authors reported that using laser-induced graphene (LIG) they obtained nearly 100% of bacteria inhibition rate under 0.75 kW/m^2^ irradiation for 10 min. Very recently Goswami et al. fabricated the functionalized graphene (fG) filter-based 3D-printed facial protective equipment [129]. The authors reported that the fG filter shows 84.00%, 92.61% and 98.20% of bacterial filtration efficiency with 20 μm, 10 μm and a melt blown filter, respectively, with lower breathing resistance values as compared to other commercialized masks. They tested an fG-coated mask against SARS-CoV-2 viral particles, showing a complete arrest of viral transmission at fG-coated layers. Modifying the mask filters with graphene has been of great interest due to its potential use as antibacterial and virucidal properties [130]. Indeed, some companies have commercialized face masks in which graphene is coated and/or embedded. As for all the nanomaterial composites, a possible limitation of this type of masks could be the leakage of nanoparticles. In fact, some authors reported that the interaction of graphene or GO nanoparticles with viable cells and biochemical can be considered unsuitable and dangerous to the human body [131]. For this reason, a challenge in GO-based medical devices is to achieve a stable immobilization of nanomaterials in the devices. The potential degradation of face masks under extreme working conditions was very recently studied by Torres et al. [130] who carried out tests complementary to the present standard tests to ensure the security of new filters based on composites or nanomaterials. 

## 6. Conclusions

Several efforts have been made by the research communities around the globe to contain and reduce the impact of COVID-19. However, the rapid spread of COVID-19 virus was not easily to reduce, and it remains very important to prevent airborne transmission with a respirator filter obtained by using innovative or existing technologies. Very interesting materials that can be used for this purpose are graphene and graphene oxide. This review, after an initial overview on application of graphene-based membrane for water purification, focuses on the antibacterial and antiviral activity of graphene-based materials and the possibility of using graphene-based membrane for reusable and recyclable masks in order to help researchers to find in graphene materials help to fight dangerous viruses and bacteria.

The increased attraction towards Graphene and graphene derivatives such as graphene oxide and reduced graphene oxide is determined by the bactericidal activity of G on several Gram-negative and -positive bacteria. G is one of the most recently developed biocidal nanomaterials for its robustness, mechanical strength, great surface area and resistance to degradation, due to its superior surface-to-volume ratio. The attachment and adherence of G to biological cells is due to their active segments, edges, and surfaces. The interaction of G-based nanomaterials with bacterial cells depends on their sheet size, surface area, roughness, hydrophilicity, dispersibility and functionalization.

Very recently the possibility was reported to make reusable masks coated on graphene that can be sterilized under sunlight irradiation where the surface temperature can quickly increase to over 80 °C. Other authors produced a prototype for a customizable/multi-used protective respirator mask in which CuO and GO nanoparticles should inactivate viral particles.

The study of graphene-based membrane with antiviral activity is still at a nascent stage that needs further work. Indeed, despite the great interest in the use of graphene to modify the mask filters due to its potential use as antibacterial and virucidal properties, additional research efforts should be direct also to the use of new methodologies to complement the actual standard tests and ensure the security of the filters modified with nanomaterials.
